# The Impact of Body Composition on Cardiorespiratory Fitness in Adult Females

**DOI:** 10.7759/cureus.55428

**Published:** 2024-03-03

**Authors:** Kavita Sudersanadas, Maha Alturki, Winnie Phillip, Aseel Al Koblan, Prachi Tambur, Sreekanth Komath Mohan, Lama Saleh Alsantali, Ghada Ibrahim Alhoumedan, Mayadah Salem Alenazi, Abeer Almudaihim

**Affiliations:** 1 College of Applied Medical Sciences, King Saud Bin Abdulaziz University for Health Sciences, Riyadh, SAU; 2 King Abdullah International Medical Research Center, Ministry of National Guard Health Affairs, Riyadh, SAU

**Keywords:** body mass index (bmi), adult females, vo2 max, body composition, cardiorespiratory fitness

## Abstract

Introduction: This study investigates the impact of body composition on cardiorespiratory fitness (CRF) in adult females, focusing on factors such as maximal oxygen uptake (VO_2_ max). It also emphasizes the importance of maintaining a physically active lifestyle for achieving CRF. Previous research links CRF to protection against metabolic syndrome.

Objective: To investigate the impact of body composition as specified by body mass index (BMI), fat-free mass (FFM), fat mass (FM), and basal metabolic rates (BMRs) on CRF in adult females.

Materials and methods: Adult females aged 19-24 participated in this prospective cross-sectional experimental study (n=110). The study excluded those with specific health conditions. Anthropometric measurements, bio-impedance analysis, and a Balke treadmill test were conducted to assess VO_2_ max and, hence, the CRF. Nutrient intake was assessed, and energy requirements were calculated. The data were analyzed using Statistical Product and Service Solutions (SPSS, version 21; IBM SPSS Statistics for Windows, Armonk, NY). The test statistics deployed were mean (± SD), ANOVA, Pearson's correlation coefficient, post-hoc Bonferroni test, and regression analysis.

Results: The study revealed significant differences in anthropometry among BMI categories. Energy intake showed no significant variation. Body mass distribution, BMRs, and vital signs significantly differed among BMI groups. Most participants exhibited poor CRF; a negative correlation between BMI and VO_2_ max was observed.

Conclusion: Body compositions, particularly BMI and FFM, body fat percentage, and BMR, influence CRF in young adult females. Poor CRF was prevalent among participants, indicating a potential impact on cardiovascular health. The findings underline the importance of addressing lifestyle factors in promoting better cardiorespiratory health among young adult females.

## Introduction

Maintaining a physically active lifestyle is crucial for achieving cardiorespiratory fitness (CRF) or endurance. Regular exercise, including aerobic exercise (AE) and resistance exercise (RE), is recommended by the American College of Sports Medicine (ACSM) to improve CRF and physical function at different ages [1]. Higher CRF is linked to favorable levels of significant cardiovascular disease (CVD) risk factors, lower prevalence and severity of subclinical atherosclerosis, and a reduced risk of developing primary and secondary clinical CVD events [2]. In physically active young men and women, subclinical atherosclerosis has an inverse association with CRF, highlighting the importance of endurance capacity in reducing the risk of early atherosclerosis [3]. Sports and leisure time-related physical activity (PA) may attenuate the age-related decline in CRF [4]. Completion of a structured exercise intervention, such as active commuting or vigorous-intensity leisure-time exercise, is associated with long-term maintenance of improvements in CRF and body composition (BC) [5].

According to Saltin's [6] definition, cardiorespiratory endurance is engaging in whole-body, large-muscle exercise at moderate-to-high intensities for extended periods [6]. Such PA was practiced by 17.4% of adults in Saudi Arabia [7]. Previous research has shown that CRF protects adults against metabolic syndrome [8].

BC and CRF are interconnected physiological parameters whose imbalances can result in metabolic derangement [9], putting the individual at risk of CVD, type 2 diabetes mellitus, and hypertension [10]. Human BC measurements refer to objective methods to assess the distribution of fat, fat-free mass, and muscle mass in the body [11]. In addition, the assessment of BC provides an insight into both nutritional status and the functional capacity of the body and quality of life [12].

The prevalence of metabolic disorders in Saudi Arabia is a significant health concern, as reported by the Global Burden of Disease study [13]. Recent studies have shown that metabolic risk factors, such as obesity, diabetes, and hypertension, are significant contributors to years lived with disability among young people in Saudi Arabia [14]. The low levels of PA and sedentary lifestyle significantly contributed to the high prevalence of metabolic syndrome among young adults in Saudi Arabia [14].

Physical activity and cardiovascular fitness in Saudi females is an essential area of study due to the high prevalence of CVD in Saudi Arabia. It was reported that, earlier, Saudi females lacked access to sports and physical education, resulting in high rates of obesity and compromised health [15]. Hence, the study was conducted to assess BC's influence on adult females' CRF.

## Materials and methods

A cross-sectional study was conducted on 110 female adults aged between 19 and 24. Participants were randomly chosen, and those with lung or cardiac diseases, physical disabilities, or taking medications that could affect their cardiovascular and respiratory systems were excluded. Before the study began, all participants provided written consent voluntarily.

Prior to the start of the study, a 24-hour recall [16] of food intake was conducted to assess the mean energy-giving nutrient intake of the subjects. The daily energy requirement was calculated by multiplying body weight by 37 kcal/kg/day [17]. Anthropometric measurements were taken using universally accepted protocols, including height, weight, waist circumference, and hip circumference [18].

The body composition of the subjects was measured using a bio-impedance analyzer (BIA 450; Biodynamics Corp., Shoreline, WA). This non-invasive procedure involved placing two electrodes on the person's right wrist and right foot to measure fat-free mass (FFM), fat mass (FM), body fat percentage (BF %), and basal metabolic rate (BMR) [19]. In addition, the analyzer provided the output related to body mass index (BMI) based on the entered height and weight of the individual participants.

The study subjects were categorized based on their BMI into three groups, namely, underweight, normal, and overweight. After taking vital signs, a Balke treadmill test [20] was conducted to measure the maximal oxygen uptake (VO_2_ max) of each participant, with respiratory rate (RR), blood pressure (BP), and heart rate (HR) being monitored throughout. Participants were asked to abstain from eating or drinking for four to five hours before the test and were informed that they could discontinue the test at any point if they felt tired.

VO_2_ max was calculated using the formula: VO_2_ max = 1.38 x T + 5.22 [21], where "T" is the total test time expressed in minutes and fractions of a minute.

The data were analyzed using Statistical Product and Service Solutions (SPSS, version 21; IBM SPSS Statistics for Windows, Armonk, NY). Categorical variables were expressed in numbers and percentages, while continuous variables were presented as mean ± SD. The study compared and assessed the correlation between the various parameters, such as anthropometric measurements, body mass distribution, vital parameters, Balke treadmill test variables, and nutrient intake with the BMI of the subjects using analysis of variance (ANOVA) and Pearson's correlation coefficient. The variables that showed statistical significance with ANOVA were further tested using the post-hoc Bonferroni test to determine which groups had significant differences. Linear regression analysis was used to predict if there was any change in VO_2_ max and body fat with the BMI of the study subjects. A p-value of less than 5% was considered statistically significant. A scatter plot was used graphically to represent VO_2_ max with BMI and body fat.

## Results

The study results indicated that the subjects had a mean height of 157.76±5.93 cm and weight was 57.88±10.92 kg, whereas the mean waist-to-hip ratio and BMI were 0.70±0.05 and 23.18±3.70 kg/m^2^, respectively. Table 1 presents the categorization of the subjects with respect to their anthropometry and nutrient intake based on nutritional status, as indicated by BMI.

**Table 1:** Categorization of anthropometry and nutrient intake of the subjects based on body mass index (BMI). ^#^Probability value as per the test for the analysis of variance (ANOVA); **highly significant; ^a^significance found in the post-hoc Bonferroni test; NA Not applicable

Particulars	Body mass index (BMI) class (kg/m^2^) (mean ± standard deviation (SD))			P value^#^
	Underweight (n=10)	Normal (n=60)	Overweight (n=40)	
Anthropometric measurements				
Height (cm)	157.2±4.51	157.2±6.40	158.8±5.50	0.376
Weight (kg)	43.8±3.12	52.9±6.20	68.9±7.69	0.001**^ a^
Waist (cm)	62.5±2.46	66.14±5.05	79.23±7.10	0.001**^ a^
Hip (cm)	86.4±2.72	94.9±5.59	107.25±6.24	0.001**^ a^
Waist-hip ratio	0.71±0.03	0.69±0.04	0.73±0.06	0.001**^ a^
Macro-nutrient intake				
Carbohydrate (g/d)	124.76±38.95	126.78±54.66	143.79±72.45	0.258
Fat (g/d)	31.47±23.99	35.98±(19.66)	39.28±23.92	0.547
Protein (g/d)	34.97±16.12	38.58±22.13)	41.04±23.06	0.708
Contribution of energy (kcal/d) from macro-nutrients				
Energy from carbohydrates (kcal)	499.02±155.79	507.11±218.65	575.17±289.79	0.358
Energy from proteins (kcal)	139.88±64.48	154.31±88.52	164.15±92.23	0.708
Energy from fat (kcal)	242.73±62.59	269.42±56.45	318.42±48.06	0.001**^ a^
Total energy intake (kcal/d)	881.63±212.84	930.84±298.44	1057.74±364.62	0.100
Recommended dietary allowance (RDA)	1620.60	1957.30	2549.30	Not applicable (NA)
% of RDA met	54.40	47.56	41.49	NA

Table 1 shows a significant difference among the anthropometry of subjects belonging to various BMI categories; however, the mean intake of energy-giving nutrients was insignificant among the subject groups. Hence, it can be assumed that the bias due to confounding factors such as high intake of energy-giving nutrients was less in this experiment, which adds more reliability to the study results. The mean energy intake of subjects was 881.63±212.84, 930.84±298.44, and 1057.74±364.62 kcal, respectively, for underweight, normal, and overweight BMI groups.

Post-hoc analysis indicated that the changes in the BMI pair of normal-underweight were highly affected by the hip circumference of the subjects, while BMI pairs of overweight-underweight were highly influenced by waist and hip circumferences (p=0.001). Overweight-normal BMI was significantly influenced by waist, hip, and waist-to-hip ratio (p=0.001). The amount of energy from fat can influence the BMI of overweight-underweight and overweight-normal pairs (p=0.001).

The subjects' mean values of BP, HR, and RR were 117.81±8.86/74.71±7.49 mm of Hg, 82.99±8.77, and 18.75±4.61 per minute, respectively. Table 2 details the body mass distribution, basal metabolic rates, and vital signs of the subjects based on their BMI.

**Table 2:** Body mass distribution and vital signs of the subjects. ^#^Level of significance as per the test for the analysis of variance (ANOVA); **highly significant; ^a^significance found in the post-hoc Bonferroni test

Variables	Underweight (n=10)	Normal (n=60)	Overweight (n=40)	P value^#^
Body mass distribution (mean ± standard deviation (SD))				
Fat-free mass (kg)	31.92±2.72	37.61±6.85	44.13±5.74	0.001**^ a^
Body fat percentage	26.97±6.95	29.94±6.27	35.38±5.34	0.001**^ a^
Fat mass (kg)	11.94±3.66	15.88±3.92	24.42±4.60	0.001**^ a^
Metabolic rate ^a ^(mean ± SD)				
Basal metabolic rate (BMR (kcal/d))	995.80±84.89	1156.50±162.39	1377.13±179.01	0.001**^ a^
Vital signs (mean ± SD)				
Systolic blood pressure (mmHg)	112.50±6.48	117.63±9.47	119.40±7.99	0.085
Diastolic blood pressure (mmHg)	70.90±5.88	74.47±8.11	76.02±6.60	0.143
Heart rate (bpm)	83.40±6.38	82.10±8.86	84.23±9.16	0.493
Respiratory rate (bpm)	17.30±4.59	18.76±4.84	19.10±4.28	0.547

**Figure 1:**
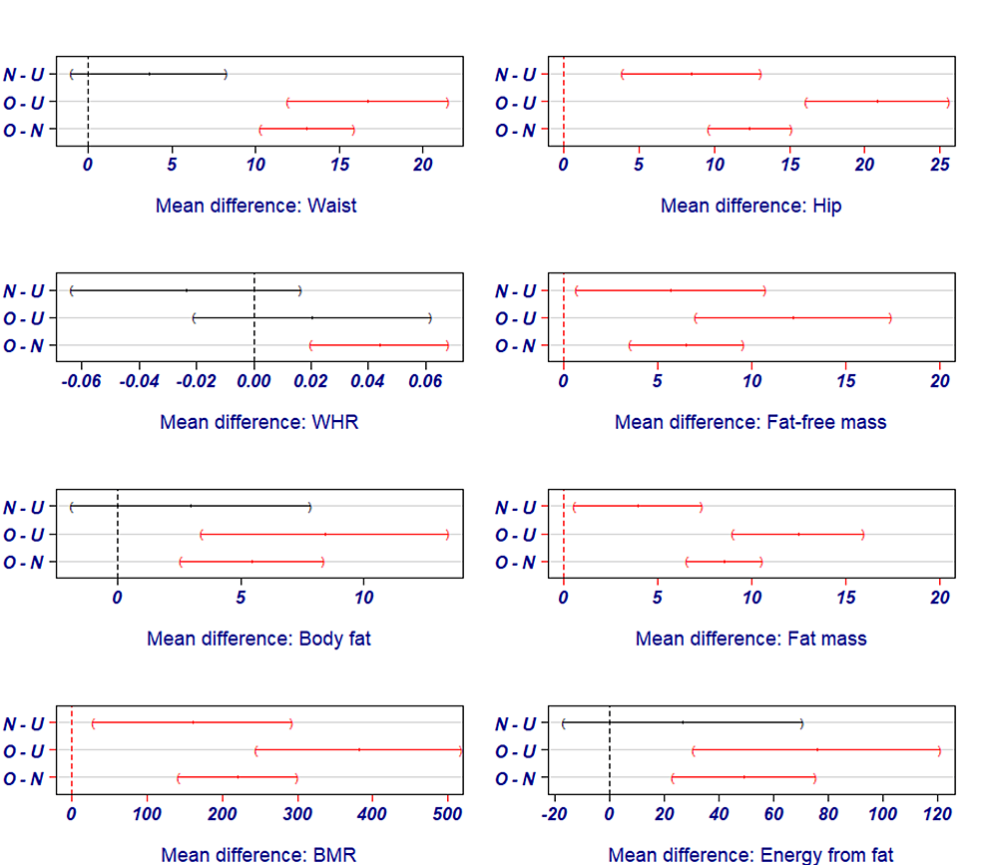
Post-hoc Bonferroni test outcome for variables influencing cardiorespiratory fitness (CRF) and body mass index (BMI). BMI pairs used in the test: N-U - Normal-Underweight; O-U - Overweight-Underweight; O-N - Overweight-Normal; WHR - Waist-to-Hip Ratio; BMR - Body Mass Index

The data in Table 2 indicate a highly significant difference in the body mass distribution and metabolic rates of the subjects of various groups, whereas the difference between the vital signs is insignificant. Post-hoc test indicates that fat-free mass, fat mass, and BMR can influence BMI of normal-underweight, overweight-underweight (O-U), and overweight-normal (O-N) BMI pairs (p=<0.05), while BF % can affect BMI pairs of O-U and O-N BMI pairs (p=0.001) (Figure 1).

Relationship between CRF and body mass distribution

The Balke treadmill test assessed the subjects' CRF (VO_2_ max); the mean VO_2_ max recorded in the sample was 25.1 mL/kg/min. Table 3 indicates the results of the Balke treadmill test.

**Table 3:** Distribution of the Balke treadmill test variables based on the body mass index (BMI). *Numbers in parenthesis indicate SD; **numbers in parenthesis indicate percentage; ^#^Test for the analysis of variance (ANOVA)

Balke treadmill test variables	BMI classification*			P value^#^
	Underweight (n= 10)	Normal (n=60)	Overweight (n=40)	
Distance walked (km)	1.40_(1.12)_	1.20_(0.93)_	0.98_(0.54)_	0.244
Test duration (min)	17.74_(13.99)_	14.89_(10.62)_	12.65_(6.42)_	0.271
Final inclination (%)	9.40_(5.48)_	8.45_(5.17)_	7.42_(3.59)_	0.388
Speed (km/h)	4.80	4.80	4.80	-
VO_2_ max (mL/kg/min)	29.70_(19.31)_	25.97_(14.41)_	22.50_(9.02)_	0.228
Classification of VO_2_ max**				Total
Poor	8_(80.00)_	45_(75.00)_	37_(92.50)_	90_(81.80)_
Fair	0	5_(8.30)_	0	5_(4.50)_
Good	1_(10.00)_	6_(10.00)_	1_(2.5)_	8_(7.30)_
Excellent	0	1_(1.70)_	1_(2.5)_	2_(1.80)_
Superior	1_(10.00)_	3_(5.00)_	1_(2.5)_	5_(4.50)_
Total	10_(100)_	60_(100)_	40_(100)_	110_(100)_

The study points out that the mean distance walked by the participants during the treadmill test and the VO_2_ max were lower for overweight subjects. However, there was no significant difference between the distance walked and VO_2_ max of subjects belonging to different BMI categories. It was also observed that the majority (81.80%) are of poor CRF as per the Cooper Institute of the VO_2_ max classification [22].

**Figure 2:**
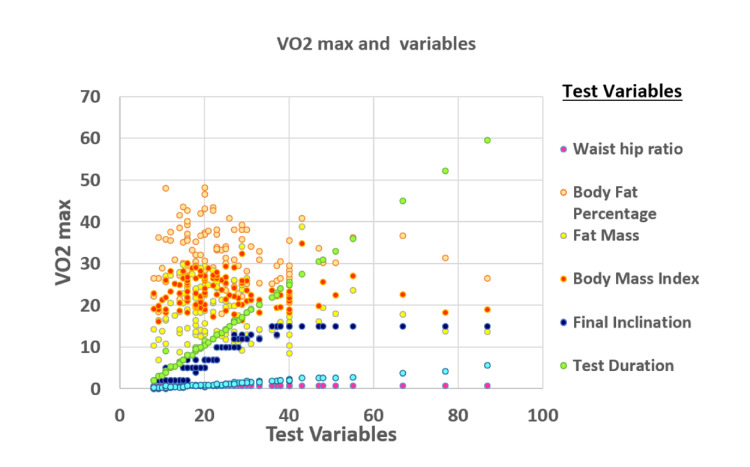
Scatter plot for VO2 max with waist-hip ratio (WHR), body fat percentage, fat mass (kg), BMI (kg/m2), final inclination (%), test duration (minutes), and the distance walked (km).

The relationship between the VO_2_ max and BMI and between the VO_2_ max and test variables are given in Figure 2.

It was observed that an insignificant negative correlation existed between the VO_2_ max and waist-hip ratio (r=-0.164; p=0.086), BMI (r=-0.112; p=0.245), BF % (r=-0.098; p=0.309), FM (r=-0.092; p=0.341), and BMR (r=-0.003; p=0.977). In addition, the VO_2_ max is positively associated with FFM. However, there was a significant positive correlation found between the VO_2_ max and Balke treadmill test variables, such as distance walked (r=0.990; p=0.001), test duration (r=0.994; p=0.001), and final inclination (r=0.841; p=0.001).

The linear regression equation (BMI=34.38-0.402 (VO_2_ max)) shows that, with one unit increase in BMI, the VO_2_ max decreases by 0.402, and with one unit increase in BF %, the VO_2 _max decreases by 0.049 (BF %=32.87-0.049 (VO_2_ max).

## Discussion

Young adult females in Saudi Arabia tend to lead a physically inactive lifestyle [23], which can increase their risk of weight gain, obesity, changes in body composition, and cardiovascular diseases [11]. To better understand this issue, a study was conducted among females aged 19-24 to observe how body composition affects cardiorespiratory endurance, measured by VO_2_ max.

The study participants were underweight or normal or overweight as per BMI and had a normal waist-to-hip ratio [24]. The mean macronutrient intake, as depicted by the 24-hour recall of food and nutrient intake, was found to be higher among the overweight subjects, but there is no significant difference between the mean intakes of normal/obese/overweight category of subjects (p=0.258; 0.547 and 0.708, respectively, for carbohydrate, fat, and protein intakes). The insignificant macronutrient intakes indicate insignificant energy intakes, and, hence, it can be assumed that the results are reliable without confounding factors such as variation in nutrient and calorie intakes. Earlier reports indicated a significant interaction between CRF and healthy dietary intake [25]. The vital signs of the subjects were also shown insignificant differences.

Basal metabolic rate (BMR) is the most critical indicator of human metabolism and undesirable health outcomes [26]. The BMR reflects a combination of cardiopulmonary function and body mass [26]. In our study, we observed that there is a significant difference between the BMR of subjects with underweight, normal, and overweight (p=0.001). It is confirmed that the BMR varies significantly with changes in weight and fat mass [9]. This variation may be due to increased body surface area, fat cells, and metabolism.

During the Balke treadmill test, the mean distance walked, the inclination and duration of walking, and VO_2_ max were less for the subjects who were overweight. This indicates that overweight individuals experienced tremendous physiological stress during the test. In addition, with inclination, the gait modifications done by the subjects adjusted their walking patterns to reduce overload on lower limb joints. These biomechanical changes in gait may contribute to the lower mean distance walked and VO_2_ max observed in overweight subjects during the Balke treadmill test. However, we did not find any significant difference between the abovementioned variables, such as the mean distance walked, the inclination and duration of walking, VO_2_ max, and different categories of BMI. The VO_2_ max is negatively correlated with waist-hip ratio, BMI, percent of body fat, fat mass, and BMR, and it is positively associated with fat-free mass, though the relationship is insignificant. Similar findings were also reported in earlier studies [27,28].

The study found that 81.80% of the subjects had poor cardio-respiratory fitness, irrespective of the BMI category and body composition. The data pointed out that none of the subjects had normal weight obesity. Normal weight obesity is indicated by a BMI range from 18.5-24.9 kg/m^2^ and with BF% ≥ 30% [29]. Hence, poor cardiovascular fitness is an indication of poor diet quality.

The subjects' diets were found to be deficient in macronutrients and energy. Carbohydrates and fats formed major energy contributors. There is a significant difference in energy contribution among BMI groups, which is more than 20% of the total energy intake, which points out that the subjects followed a high fat, low protein, and moderate carbohydrate diet. Consuming insufficient calories and relying heavily on fat or energy can reduce the metabolic rate as the body attempts to conserve energy, resulting in poor CRF. Such diets did not have a superior effect on BC and CRF [30].

According to the linear regression equation, an increase of one unit in BMI results in a decrease of VO_2_ max by 0.402, and an increase of one unit in BF % leads to a decrease of VO_2_ max by 0.049. The equation is as follows: BMI=34.38-0.402 (VO_2_ max) and BF %=32.87-0.049 (VO_2_ max).

Among the variables of body composition, BF % has a significant influence on the BMI pairs of O-U and O-N BMI pairs of the post-hoc tests. In addition, the mentioned BMI pairs were further highly influenced by the amount of energy consumed from fat, FFM, FM, and BMR, which in turn can affect the VO_2 _max or CRF.

Study limitations

The study was conducted among healthy young adults aged 19-24 with underweight/normal/overweight BMI. The study with different age groups and subjects with obesity and different body compositions may provide a better scenario of the relation between body composition and CRF or VO_2_ max.

## Conclusions

The study shows healthy young adult females have a poor VO_2_ max. The subjects' CRF is influenced by the contribution of energy from fatty food and body composition, as determined by the FFM, FM, and BMR. Physiological stress and gait changes due to being overweight impacted the mean distance walked, inclination, duration of walking, and VO_2_ max during the Balke treadmill test of the subjects who were overweight. BMI and BF % can be used to determine the CRF. It is essential to prioritize healthy lifestyle choices to boost cardiorespiratory health in young women. The study emphasizes the need to take action toward promoting better health for females in their early adulthood. The study recommends further studies with different age groups and subjects with obesity and varied body composition.
